# Supplementary material for: Effectiveness and Safety of Ayurvedic Medicines in Type 2 Diabetes Mellitus Management: A Systematic Review and Meta-Analysis
Source: Front Pharmacol. 2022 Jun 8;13:821810. doi: 10.3389/fphar.2022.821810 (PMC9213670; doi:10.3389/fphar.2022.821810)
Supplement: Supplementary file 1 [file DataSheet1.zip › Supplementary Material/Forest and Funnel Plots/Plantago ovata/HbA1c.pdf]

Plantago ovata - HbA1c

Study	Intervention N	Intervention Mean	Intervention SD	Comparator N	Comparator Mean	Comparator SD	Mean Diff. with 95% CI	Weight (%)
Ziai 2005	21	8.90	0.92	15	10.50	2.32	-1.60 [-2.69, -0.51]	16.51
Feinglos 2013	26	7.29	0.41	8	7.75	0.40	-0.46 [-0.78, -0.14]	42.48
Abutair 2016	18	7.50	0.54	18	8.50	0.56	-1.00 [-1.36, -0.64]	41.01
**Overall**							-0.87 [-1.41, -0.33]	

Heterogeneity: $\tau^2 = 0.15$, $I^2 = 74.11\%$, $H^2 = 3.86$

Test of $\theta_i = \theta_j$: Q(2) = 7.28, p = 0.03

Test of $\theta = 0$: z = -3.15, p = 0.00

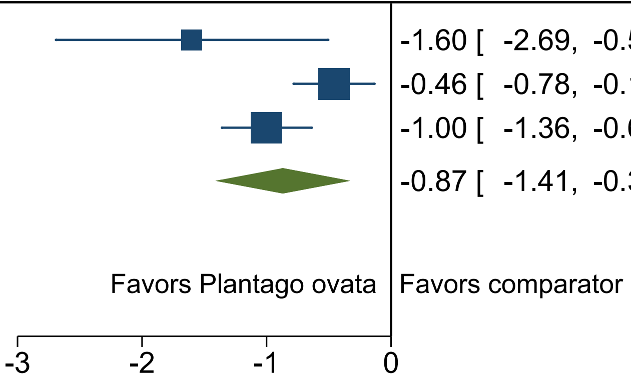

Random-effects REML model
